# Clinical safety assessment of implanted brain-computer interfaces: current evidence and a pathway to standardization

**DOI:** 10.3389/fnins.2026.1994636

**Published:** 2026-10-28

**Authors:** Baozhou Xing, Li Zhang, Wen-Jun Tu

**Affiliations:** 1 Clinical Medical College of Shandong Second Medical University, Weifang, China; 2 Department of Neurology, Aerospace Center Hospital, Peking University Aerospace School of Clinical Medicine, Beijing, China

**Keywords:** adverse events, clinical safety, implanted brain-computer interface, longitudinal assessment, neurosurgery, standardisation

## Abstract

Implanted brain-computer interfaces are beginning to move beyond short laboratory sessions into long-term and home use. The evidence used to judge clinical safety remains fragmented. This review examines penetrating intracortical, cortical-surface (including subpial) and cerebral-venous systems, with stimulation considered as an additional exposure. Published studies report perioperative neurological and wound complications, vascular patency, stimulation-related effects, changes in devices and materials, and loss of functions on which participants may depend. Comparison across architectures remains difficult because cohorts are small and sometimes overlap, exposure denominators vary, long-term surveillance is incomplete, and retrieval data are limited. Chronic safety cannot be inferred from one test. Clinical review, problem-directed imaging, device interrogation and participant-level outcomes capture different parts of the safety record and need to be read together over time. We use this evidence to set out a lifecycle framework spanning the preimplant baseline, implantation, recovery, chronic use, new findings and end-of-use. It combines a small common dataset with architecture-specific modules and an event-triggered route for confirmation, attribution, escalation and outcome recording. Before this approach can inform formal guidance, it requires prospective multicentre testing, independent adjudication, input from participants and caregivers, registry follow-up and periodic revision. The near-term task is to make safety evidence comparable and clinically useful without treating any implanted BCI architecture as generally safe.

## Introduction

Implanted brain-computer interfaces link neural activity directly to an external device, and some also return sensory information through cortical stimulation. Their clinical aim is to restore communication or control for people with severe motor impairment. Intracortical recordings have enabled cursor and robotic-limb control (Hochberg et al., 2006; Hochberg et al., 2012; Collinger et al., 2013; Gilja et al., 2015), followed by typing, communication and brain-controlled muscle stimulation (Jarosiewicz et al., 2015; Pandarinath et al., 2017; Bouton et al., 2016; Ajiboye et al., 2017). Speech decoding, handwriting and tactile feedback have broadened the functional range (Card et al., 2024; Flesher et al., 2021; Moses et al., 2021; Willett et al., 2021). Tablet control, wireless operation and multiyear home use have also moved selected systems beyond closely supervised laboratory sessions (Nuyujukian et al., 2018; Simeral et al., 2021; Vansteensel et al., 2024; Card et al., 2026). Taken together, this work is beginning to move implanted BCIs beyond signal-decoding demonstrations. A system may now remain in place for years and become part of a person’s daily means of communication or action.

The apparent size of the field depends on what is counted: publications, implanted participants and independent trials are not interchangeable. A review restricted to chronic clinical trials conducted from 1998 to 2023 identified 28 trials, 21 research groups, 67 implanted participants and 165 peer-reviewed publications (Patrick-Krueger et al., 2025). A broader review included 93 studies and 214 patients because it used different eligibility boundaries (Lim et al., 2025). Among 112 motor-BCI studies, 49.1% were published from 2020 onwards, but only 17.9% assessed clinical outcomes (Dohle et al., 2025). Functional performance has moved faster than the clinical evidence needed to interpret harm, especially during long-term use.

The evidence gap is clearest when safety is followed beyond the operation. BrainGate recorded 68 device-related adverse events during 12,203 participant-days in 14 participants (Rubin et al., 2023). SWITCH provided 12-month vascular and clinical observations in four implanted participants (Mitchell et al., 2023), while a fully implanted cortical-surface system was followed through years of home use (Vansteensel et al., 2024; Davis et al., 2025). A long-term intracortical-microstimulation report added 27 combined implant-years and more than 170 million stimulation pulses in five participants (Greenspon et al., 2026). These studies are informative, but their architectures, exposures and outcome sets differ. Biological evidence is similarly uneven: explant and post-mortem studies reveal material or local tissue changes only at selected endpoints (Bjånes et al., 2025; Woeppel et al., 2021; Szymanski et al., 2021), whereas animal experiments examine mechanisms under conditions that do not supply a human incidence rate (Franklin et al., 2023; Wellman et al., 2025; Joseph et al., 2021; Patel et al., 2023). A successful decoding session, a stable impedance trace or an uneventful clinic visit addresses only one part of the safety question.

Several reviews have described the technological, neurosurgical and ethical setting of implanted BCIs (Shen et al., 2023; Banta et al., 2026; Vansteensel et al., 2023; Ienca et al., 2025). Evidence from deep brain stimulation and invasive epilepsy monitoring helps to frame adjacent surgical and device risks, but it cannot be treated as a BCI event denominator (Bullard et al., 2020; Rasiah et al., 2023; Cheyuo et al., 2024; Muthiah et al., 2025). FDA guidance supplies a starting point for device development and clinical investigation (U.S. Food and Drug Administration, 2021). The gap is a prospectively validated, BCI-specific clinical pathway for moving from a new symptom, imaging finding or technical change to confirmation, attribution, escalation, action and outcome. A shared longitudinal dataset is also lacking; without it, clinical events, tissue or vascular observations, device state, functional dependence and end-of-use information cannot be interpreted consistently across programmes.

Against this background, the review considers safety evidence for penetrating intracortical, cortical-surface (including subpial) and cerebral-venous systems; stimulation is treated as an additional exposure. The discussion begins with what clinical safety needs to include, then moves through architecture-specific risks, lifecycle follow-up and the limits of current clinical, imaging and device measures. Human cohorts are placed alongside retrieval, post-mortem and animal evidence because each resolves a different part of the problem. On that basis, the final sections outline a common longitudinal core, architecture-specific modules and event-triggered review as a testable route towards standardisation. The proposal is intended to organise evidence. It is not a clinical guideline and does not prescribe universal visit intervals or treatment thresholds.

### Review approach

Literature was identified through targeted PubMed searches and reference checking. No prespecified lower publication-date limit was applied, and searches for the original review were completed on 31 August 2026. PubMed Central (PMC) and publisher websites were used to retrieve available articles. Regulatory guidance and standards were consulted through the websites of the US Food and Drug Administration (FDA) and the International Organization for Standardization (ISO). Search concepts included implanted or invasive brain-computer interfaces, neural interfaces, safety, adverse events, surgical complications, tissue and vascular responses, device failure, explantation and long-term follow-up. These concepts guided topic-specific searches rather than a single exhaustive search strategy.

Studies were selected for their relevance to the safety of implanted BCIs. Human reports informed the assessment of clinical events, device exposure and longitudinal outcomes. Animal experiments, explant analyses and post-mortem studies were included to examine biological or material changes that could not be measured directly in living participants. Relevant reviews provided context and additional primary references. The evidence was organised by implant architecture and assessment domain, with overlapping participant cohorts identified where the reports allowed this. Differences in event definitions and exposure denominators precluded pooling incidence estimates.

## What does safety mean for an implanted BCI?

Serious adverse events alone do not capture the whole implant experience. Hardware complications and neural-interface reporting work distinguish clinical consequences from technical failure (Bullard et al., 2020; Eiber et al., 2021). Participant-preference studies emphasise independence, usability and support (Brannigan et al., 2024b; Blabe et al., 2015; Huggins et al., 2011). Performance is not a safety measure in itself. However, interruption of a relied-upon function becomes a safety concern when it risks clinically meaningful harm or leaves no effective fallback. Investigational-device ethics guidance addresses the associated responsibilities (van Stuijvenberg et al., 2026).

Figure 1 groups these observations into four domains: clinical harm, tissue or vascular state, technical integrity, and consequences of interrupted function. This division is analytical rather than an established taxonomy, but it is useful because findings across the domains may not change together. An explant study examined 980 microelectrodes from 10 arrays removed from three people after 956–2,130 days, together with one unimplanted control array, and found material damage whose relation to electrical performance differed by electrode type (Bjånes et al., 2025). Conversely, signal instability or recalibration can occur without evidence that tissue injury is the cause (Colachis et al., 2021; Wilson et al., 2026; Pun et al., 2024; Swanson et al., 2026).

**Figure 1:**
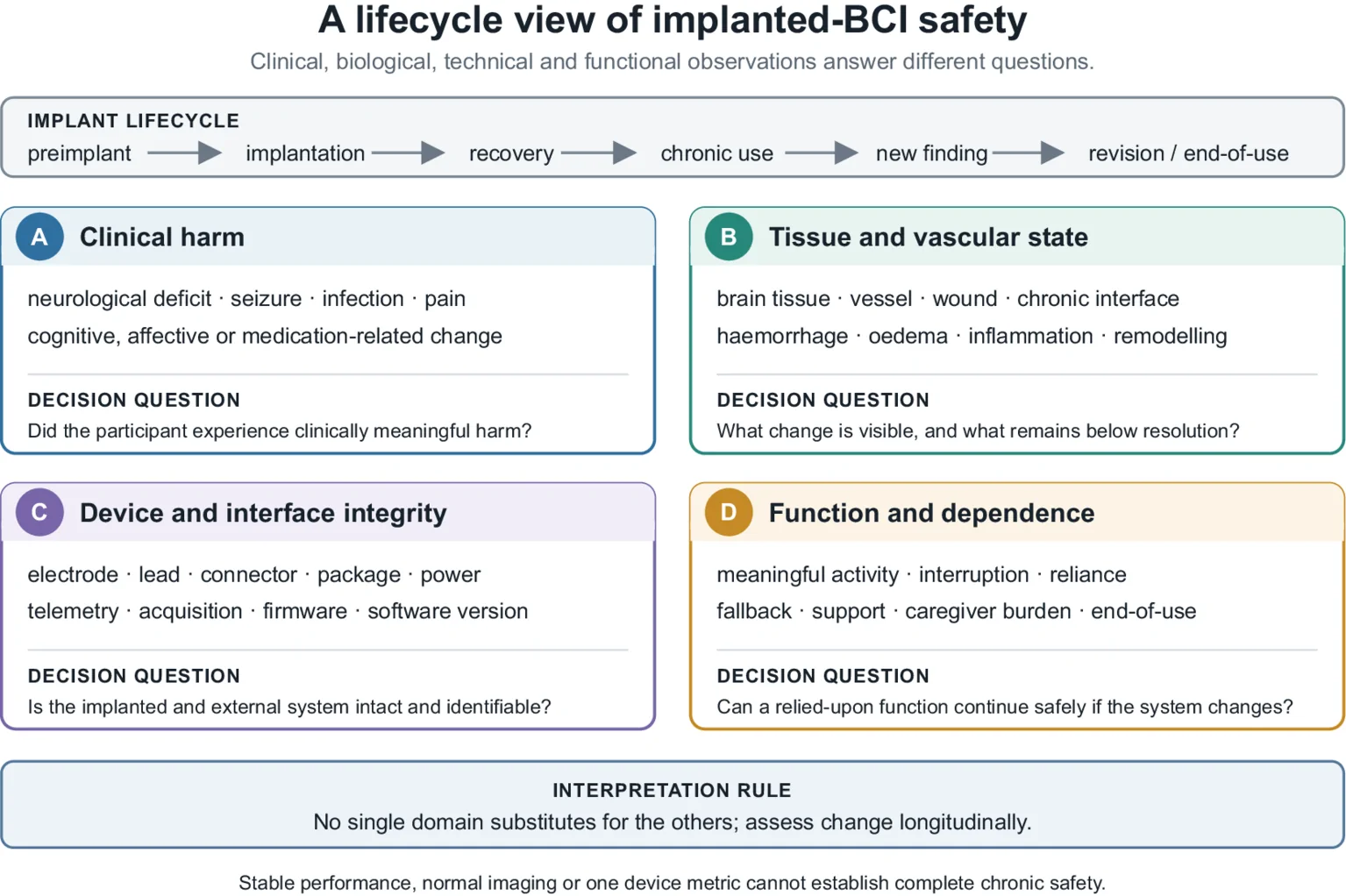
Four complementary observation domains across the implant lifecycle. Interruption of a relied-upon activity is a safety concern when it risks clinically meaningful harm or leaves no effective fallback.

## Implant architecture determines the main safety priorities

The anatomical compartment and access route determine which tissues contact the device, which complications remain plausible and which observations are available during follow-up (Shen et al., 2023; Bullard et al., 2020; U.S. Food and Drug Administration, 2021). Figure 2 maps those differences across the main interfaces.

**Figure 2:**
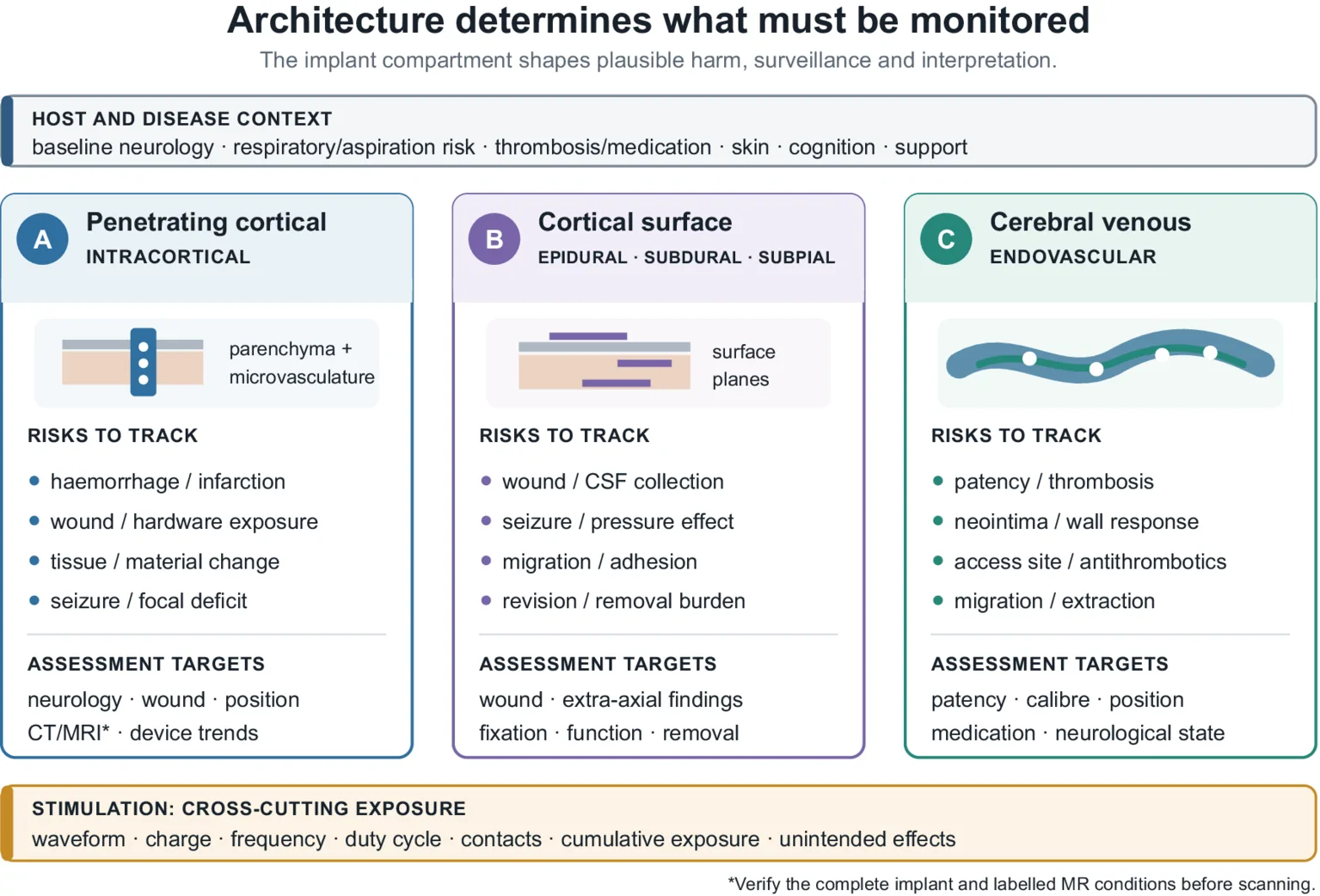
Architecture-specific priorities for clinical safety assessment. Major risks and assessment targets are shown by interface; stimulation is recorded as a cross-cutting exposure.

### Penetrating intracortical systems: parenchyma, microvasculature and the chronic access route

Intracortical electrodes enter neural tissue and may injure small vessels. Implantation reports describe the importance of technique, position and cortical targeting (House et al., 2006; Ikegaya et al., 2025; Downey et al., 2024). Perioperative assessment centres on haemorrhage, infarction, oedema, seizure and new deficit. A percutaneous connector also creates a chronic skin interface (Wagner et al., 2026). Neuromodulation infection guidance provides adjacent clinical context, but is not a BCI-specific incidence estimate (Pritzlaff et al., 2025).

From 2004 to 2021, BrainGate enrolled 14 implanted participants, with a mean implant duration of 872 days and 12,203 participant-days of safety follow-up (Rubin et al., 2023). Investigators recorded 342 adverse events; 274 (80.1%) were unrelated to the investigational device, while 68 were device-related and six of those were serious (Rubin et al., 2023). Incision or pedestal-site irritation and related skin changes accounted for 35 of the 68 device-related events. Across 17,927 array-implant-days, no intracranial or deep-tissue device-related infection required hospitalisation, intravenous antibiotics or explantation (Rubin et al., 2023). These figures describe event counts from one percutaneous platform, not the proportion of participants who experienced each event.

These events are also a reminder that not every risk can be assigned to the device itself. Two participants with previous traumatic brain injury had seizures on postoperative day 4 after antiseizure prophylaxis was absent or interrupted; neither had further seizures or lost the ability to continue in the trial (Rubin et al., 2023). A participant with previous pulmonary embolism developed recurrent embolism on postoperative day 3 while therapeutic anticoagulation was still withheld and recovered after it was restarted (Rubin et al., 2023). These cases connect the event phenotype with baseline risk, perioperative medication and later outcome.

The chronic interface remains largely hidden during use. The 980-electrode human explant analysis documented coating, insulation, shaft and tip changes after 956–2,130 days; 1-kHz impedance tracked several damage measures for SIROF electrodes, but the relation was not uniform across materials or arrays (Bjånes et al., 2025). Other retrieval and post-mortem reports describe material findings and local neuropathology at selected endpoints (Woeppel et al., 2021; Szymanski et al., 2021). Animal studies examine inflammasome activation, mural-cell changes and glial responses (Franklin et al., 2023; Wellman et al., 2025; Joseph et al., 2021). Histological and multi-omic studies further characterise encapsulation, scarring and inflammation (Patel et al., 2023; Darlot et al., 2024; Druschel et al., 2026; Huff et al., 2026). Preclinical studies show that connector, insulation, biological and unknown failure modes can coexist (Barrese et al., 2013; Barrese et al., 2016; Nolta et al., 2015; Chen et al., 2023). Polyimide and multifunctional-material studies further show that interface response depends on design and material context (Orlemann et al., 2026; Yao et al., 2026). This work helps to explain plausible mechanisms and measurement blind spots, but it does not provide a contemporaneous clinical test for the onset of injury or a human incidence threshold.

### Cortical-surface and subpial systems: chronic interface and removal considerations

Surface arrays reduce or avoid multiple penetrating electrode tracks. The trade-off is a different chronic risk profile, not necessarily a lower one. Epidural, subdural and subpial placements differ in dural opening, cortical contact, fixation and removal plane (Shen et al., 2023). Subdural-grid epilepsy studies identify infection, haemorrhage and cerebrospinal-fluid complications as adjacent concerns, although their exposure differs from chronic BCI use (Arya et al., 2013).

Long-term surface-system evidence is still built from individual cases and small programmes. One fully implanted subdural ECoG system remained usable at home for 5 years in a participant with spinal cord injury; electrode impedance stabilised within 6 months and the battery remained at an operable voltage (Davis et al., 2025). A separate Utrecht system was followed for 7.5 years (Vansteensel et al., 2024). Earlier reports describe fully implanted subdural communication and epidural WIMAGINE recording and exoskeleton control (Vansteensel et al., 2016; Benabid et al., 2019; Larzabal et al., 2021; Moly et al., 2022). Switch-scan spelling, speech and streaming voice studies add functional observations (Candrea et al., 2024; Metzger et al., 2023; Littlejohn et al., 2025), and nighttime use has been examined in late-stage amyotrophic lateral sclerosis (ALS) (Leinders et al., 2026). Two newer 1,024-channel reports concern short intraoperative use rather than multiyear implantation (Hettick et al., 2026; Lehner et al., 2026).

The Utrecht participant used the fully implanted system independently at home for almost 7 years. For more than 3 years it was her only way to initiate communication; use declined after about year 6 and ended 7.5 years after implantation when control became unreliable, although no technical malfunction was identified (Vansteensel et al., 2024). End-of-use, fallback and dependence therefore remained clinically relevant even without a conventional device failure.

### Cerebral-venous systems: patency, wall response and antithrombotic treatment

A cerebral-venous BCI places the chronic interface within a vein. First-in-human experience and endovascular reviews identify access-site injury, patency, thrombosis or stenosis, and migration as key questions (Oxley et al., 2021; Hosoo et al., 2026; Ognard et al., 2025). Post-stroke endovascular work adds context for patient selection and antithrombotic exposure (Brannigan et al., 2024a). SWITCH enrolled five people and implanted four. At 3 and 12 months, CT venography showed no target-vessel occlusion or thrombosis; the mean absolute change in distal-electrode position was 0.45 mm (SD 0.28) (Mitchell et al., 2023). The protocol used dual antiplatelet therapy for 3 months and aspirin through month 12. During follow-up, eight mild adverse device effects comprised four incision-site bruising events, two anaemia events, one jugular access-site haematoma and one episode of postoperative fatigue; all resolved without intervention (Mitchell et al., 2023). This four-person, 12-month record cannot determine late wall response, chronic removability or the safety of a different antithrombotic regimen.

Sheep studies from the same platform found neointimal coverage and non-uniform vascular remodelling despite stable impedance (Opie et al., 2016; John et al., 2022). These findings support separate recording of patency, calibre or wall response, position, access-site events, neurological outcome and actual antithrombotic exposure during human follow-up. The animal findings help frame these questions but do not supply a human event rate or treatment threshold.

### Stimulation is an additional exposure across architectures

Somatosensory and visual intracortical stimulation studies test different targets and percepts (Flesher et al., 2016; Fernández et al., 2021). Electrical-injury literature and neurostimulator standards make waveform, charge, frequency, contacts and duty cycle relevant to assessment (Cogan et al., 2016; International Organization for Standardization, 2017). A recent long-term human ICMS report followed five participants for 2–10 years, totalling 27 implant-years, 623 stimulation-hours and more than 170 million pulses (Greenspon et al., 2026). Fifty-three brief sensations that continued after the stimulation train were recorded as ICMS-related adverse events, with 3–25 events per participant and a mean across participants of approximately 23,000 stimulation trials per event (Greenspon et al., 2026). Most lasted less than 10 s, the longest lasted about 9.5 min, none was painful and none required medical intervention. Detection thresholds increased by an average of 3.67 μA per year, with substantial variation across electrodes and participants; ICMS still evoked tactile sensations in the participant followed for 10 years (Greenspon et al., 2026). Macaque chronic-stimulation and mouse longitudinal-imaging studies address specified regimens and different biological endpoints (Rajan et al., 2015; Suematsu et al., 2026). The human study used intermittent, closely monitored sessions with predefined stimulation limits and stopping rules. These data do not define a safe exposure for continuous real-world stimulation or for another cortical target.

The imbalance is similar across architectures. Clinically apparent events are recorded most clearly around implantation or in small prospective cohorts (Rubin et al., 2023; Mitchell et al., 2023; Davis et al., 2025; Greenspon et al., 2026). Chronic biological and material information is often obtained only at retrieval, post-mortem examination or in animals (Bjånes et al., 2025; Woeppel et al., 2021; Szymanski et al., 2021). Long-term functional reports fill another part of the record. The evidence streams are not interchangeable, and event counts alone cannot establish long-term safety.

A more useful way to read the literature is to reconstruct each safety decision: what changed, in which device and software version, after how much exposure, how the change was detected, whether it reached the participant, what response followed and what the final status was. Neural-interface reporting recommendations already support preserving the technical context (Eiber et al., 2021). A clinical standard would extend that record to interpretation and escalation.

## Safety assessment across the implant lifecycle

A practical way to follow long-term safety is to keep one continuous record from preimplant assessment through surgery, recovery and chronic use, adding further assessment when a new event occurs (U.S. Food and Drug Administration, 2021; Eiber et al., 2021; U.S. Food and Drug Administration, 2022; International Organization for Standardization, 2026). Figure 3 locates these phases within one record while leaving visit timing to the device, protocol and clinical problem.

**Figure 3:**
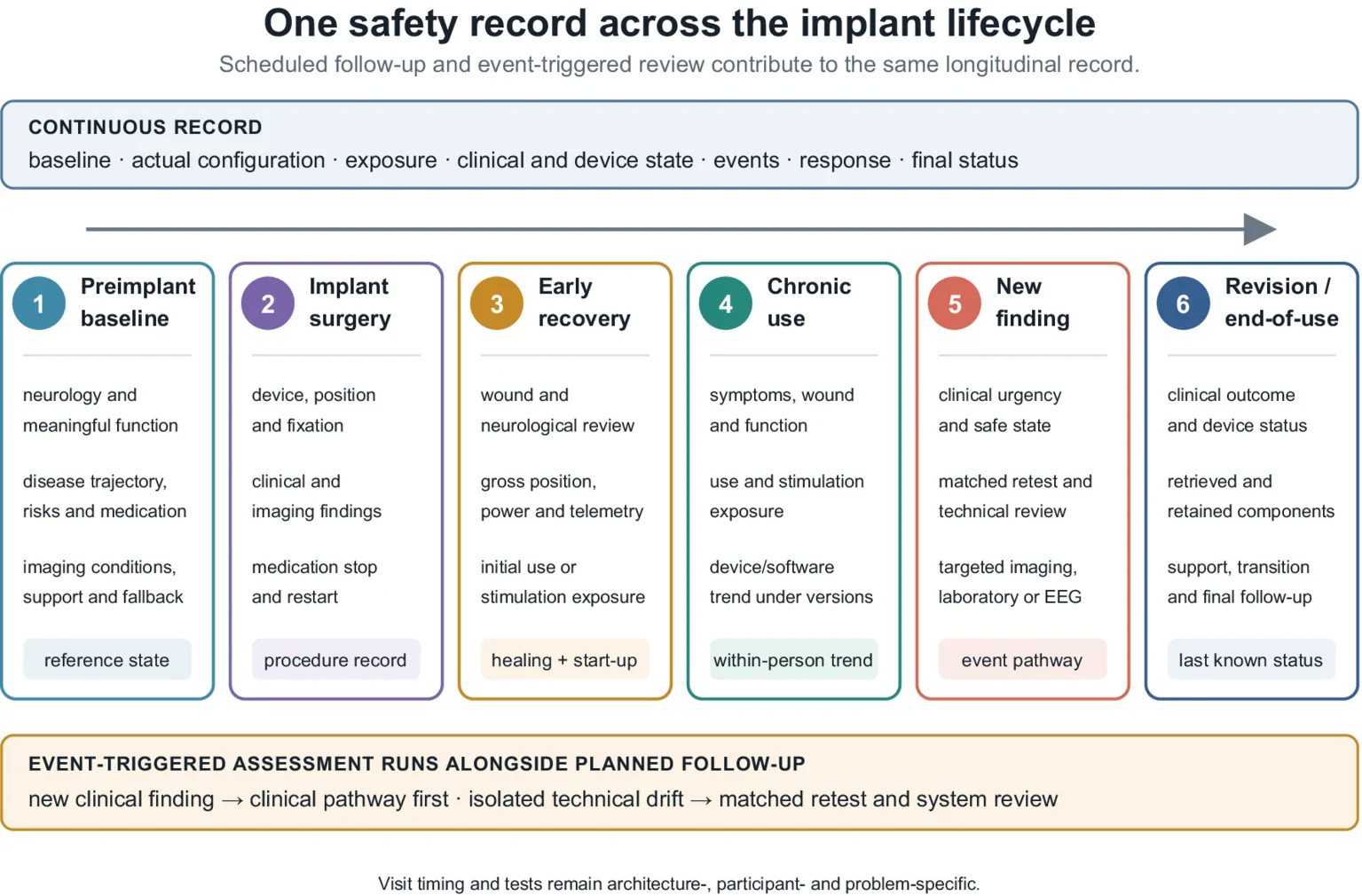
Lifecycle structure for longitudinal safety reporting and surveillance. Planned follow-up and event-triggered assessment contribute to one continuous record.

### Before implantation: establish a baseline that can explain later change

Baseline assessment preserves a reference for later change. FDA BCI guidance and late-stage ALS analyses support attention to neurological status, disease course, clinical risks and practical follow-up constraints (Vansteensel et al., 2023; U.S. Food and Drug Administration, 2021). Cognitive and affective effects described in focal neuromodulation are adjacent evidence, not established BCI risks (Wiseman et al., 2024). Within the proposed lifecycle framework, the baseline record would include relevant seizure, infection, respiratory, skin and antithrombotic history alongside communication, usual assistive technology and caregiver support. The MR-conditional status of the complete implanted system and an effective fallback would also be documented before implantation.

### Perioperative period: first determine whether immediate harm has occurred

The early priority remains vital and neurological status, bleeding, ischaemia, seizure, wound and medication complications. Deep brain stimulation (DBS) and invasive epilepsy-monitoring studies inform the surgical context (Rasiah et al., 2023; Cheyuo et al., 2024; Muthiah et al., 2025). BrainGate and SWITCH provide direct BCI event records (Rubin et al., 2023; Mitchell et al., 2023). Longitudinal interpretation requires the configuration actually implanted, its position and fixation, and any interruption or restart of anticoagulant or antiplatelet treatment to remain in the record. This reporting requirement does not imply a common medication schedule.

### Early recovery: healing, early neurological events and system start-up

During the first days and weeks, review focuses on healing, infection, seizure, oedema or new neurological change, together with gross position, power and telemetry (Rubin et al., 2023; Mitchell et al., 2023). For venous systems, early recovery assessment would include protocol- and problem-directed patency assessment together with a record of actual antithrombotic use (Oxley et al., 2021). If stimulation begins, the proposed record would capture the delivered settings and any unexpected sensation, movement, pain or electroclinical effect (Greenspon et al., 2026; Greenspon et al., 2025).

### Chronic use: follow trends rather than isolated snapshots

Chronic review benefits from repeated observations of symptoms, wound and implant-site condition, system version, use and reliance. Neural-interface reporting recommendations support preserving device and exposure information (Eiber et al., 2021). Long-term performance and recalibration studies help interpret signal and channel trends (Colachis et al., 2021; Wilson et al., 2026; Pun et al., 2024; Simeral et al., 2011). Stimulation requires its own exposure record rather than implant duration alone (Greenspon et al., 2026; Cogan et al., 2016). A persistent change would be a trigger for reassessment without presuming tissue injury.

### Event-triggered assessment alongside planned follow-up

New focal deficit, seizure, wound breakdown, fever or altered consciousness requires clinical assessment without waiting for a research visit (U.S. Food and Drug Administration, 2021; Pritzlaff et al., 2025). Within the proposed pathway, isolated impedance, channel, decoding or telemetry changes would first be retested under matched conditions, with reference, connections, power and software checked (Eiber et al., 2021; Wilson et al., 2026; Pun et al., 2024; Swanson et al., 2026). Persistent or unexplained changes would prompt clinical review because technical checks cannot exclude tissue injury (Figure 4).

**Figure 4:**
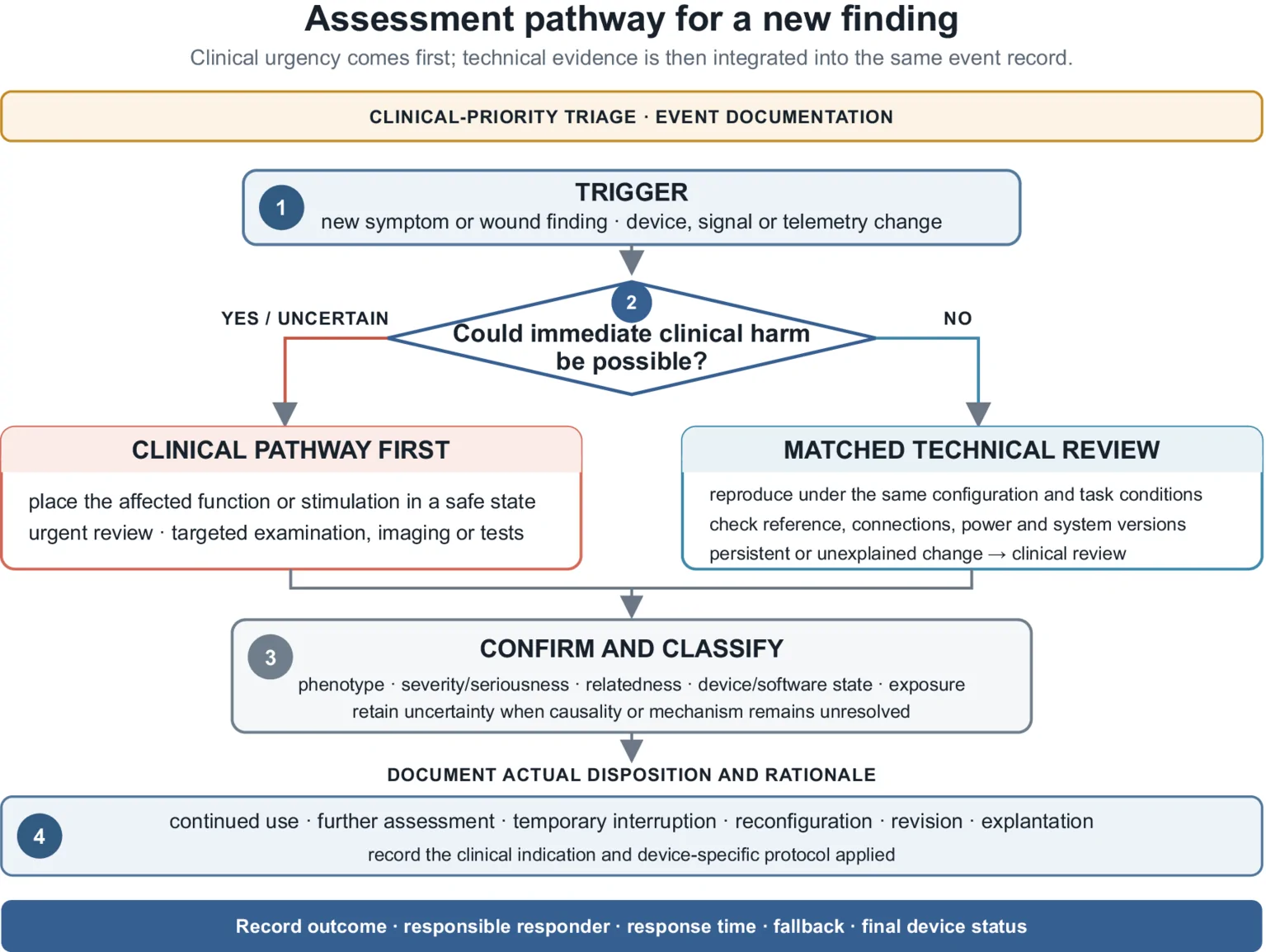
Assessment and documentation pathway for a new finding. The pathway organises review and escalation; it is not a validated treatment algorithm. Clinical urgency takes priority, isolated technical change requires confirmation, and disposition follows clinical indication and the device-specific protocol.

### Revision, explantation and end-of-use are part of follow-up

Research completion, withdrawal, loss of support, upgrade, infection or hardware failure may lead to revision, explantation or inactive retention. Retrieval has its own dissection plane, possible retained components and follow-up needs (Rubin et al., 2023; Bjånes et al., 2025; Woeppel et al., 2021). Legal and ethical work distinguishes removal from continued or discontinued access to a device (Bublitz and Gilbert, 2023; Vooijs et al., 2025). Consent, post-trial responsibility and abandonment studies support advance discussion of clinical and technical support, fallback, removal options and transition costs (Young et al., 2026; Higgins et al., 2026; Okun et al., 2024). They do not establish a universal legal rule.

## What the main assessment tools can and cannot show

Each assessment method sees only part of the safety picture. Table 1 sets out the question each method can answer and what remains outside its view.

**Table 1:** Main assessment modalities used in implanted-BCI follow-up and their interpretive limits.

Modality	Primary safety question	Main limitation	Examples
Clinical / wound / event review	Has the participant developed a neurological, infectious, wound or functional consequence?	Cannot directly show microscopic tissue, vessel-wall or material change	Rubin et al. (2023); U.S. Food and Drug Administration (2021); Pritzlaff et al. (2025)
CT / MRI	Is there haemorrhage, oedema, infection, mass effect or gross position change?	Configuration and artefact constraints; normal imaging is not microscopic stability	U.S. Food and Drug Administration (2021); Boutet et al. (2020); Harwood et al. (2024); Bezchlibnyk et al. (2024)
Vascular imaging	Is the device-bearing venous segment patent and position stable?	Segment-, protocol- and medication-dependent; limited late human data	Mitchell et al. (2023); Hosoo et al. (2026); Oxley et al. (2021); Ognard et al. (2025)
Device / electrophysiology	Has the system changed or developed a technical fault trend?	Sensitive but non-specific; acquisition, hardware and software matter	Eiber et al. (2021); Colachis et al. (2021); Wilson et al. (2026); Simeral et al. (2011)
Diagnostic EEG	Is there electrographic seizure activity or afterdischarge?	Problem-directed clinical test, not universal screening	Rubin et al. (2023); Greenspon et al. (2026)
Function / participant report	Has interruption changed meaningful activity, dependence or burden?	Confounded by disease progression and support context	Brannigan et al. (2024b); Blabe et al. (2015); Huggins et al. (2011); U.S. Food and Drug Administration (2022)
Explant / pathology	What tissue and material findings are present at retrieval?	Selected endpoints; cannot estimate longitudinal incidence or timing	Bjånes et al. (2025); Szymanski et al. (2021); Woeppel et al. (2021)

### Clinical assessment and event recording

Neurological examination, wound review, infection assessment and participant report identify clinically apparent problems: deficits, seizure-related symptoms, pain, wound breakdown, infection and disruption of daily function. They do not directly reveal microscopic tissue, vessel-wall or material changes (Rubin et al., 2023; U.S. Food and Drug Administration, 2021; Pritzlaff et al., 2025).

### CT, MRI and vascular imaging

Structural imaging addresses haemorrhage, oedema, mass effect or displacement, with modality chosen for the clinical question. Magnetic resonance imaging (MRI) adds soft-tissue information, but electrodes, leads, connectors, packaging, retained components and device state must match configuration-specific conditions (Boutet et al., 2020; Harwood et al., 2024; Bezchlibnyk et al., 2024; International Organization for Standardization, 2018). FDA guidance addresses MR testing and labelling (U.S. Food and Drug Administration, 2023). A normal study does not exclude abnormalities below its sensitivity or resolution. For venous implants, follow-up imaging should assess the device-bearing segment; SWITCH used computed tomography (CT)-based assessment of patency and position (Mitchell et al., 2023). Animal wall-remodelling findings cannot be inferred from a patent human vessel alone (Opie et al., 2016; John et al., 2022).

### Device, electrophysiological and participant-level measures

Impedance, channel yield, signal quality, stimulation threshold, telemetry and power behaviour are well suited to trend detection but are not specific for tissue injury. Comparisons require matched acquisition, reference, hardware and software conditions (Eiber et al., 2021; Colachis et al., 2021; Wilson et al., 2026; Simeral et al., 2011). Signal or recalibration changes may still arise from several technical states (Pun et al., 2024; Swanson et al., 2026). Diagnostic electroencephalography (EEG) remains a separate clinical test when seizure, afterdischarge or altered consciousness is suspected. Participant function adds another safety dimension: interruption of a relied-upon activity, new dependence and caregiver burden can matter even when the technical fault is recoverable (Brannigan et al., 2024b; Blabe et al., 2015; Huggins et al., 2011; U.S. Food and Drug Administration, 2022). This burden should be recorded alongside functional outcome and trial design fields (van Stuijvenberg et al., 2026; Sawyer et al., 2026b; Sawyer et al., 2026a).

## What can be concluded across implant architectures?

No available evidence stream covers every safety domain. BrainGate and SWITCH show that prospective follow-up can capture clinically apparent, wound, access-site and medication-related events (Rubin et al., 2023; Mitchell et al., 2023). Long-term surface reports show that selected systems can remain useful (Vansteensel et al., 2024; Davis et al., 2025), and ICMS work quantifies a defined stimulation exposure (Greenspon et al., 2026). They fill different parts of the record, but they do not establish which architecture is safest.

Function, technical integrity and biological state do not necessarily change together. Retrieval and post-mortem findings can reveal material or local tissue change after useful recording has persisted (Bjånes et al., 2025; Woeppel et al., 2021; Szymanski et al., 2021). Recording instability and recalibration, by contrast, can reflect several technical states (Colachis et al., 2021; Wilson et al., 2026; Pun et al., 2024; Simeral et al., 2011) and do not by themselves establish tissue injury (Swanson et al., 2026). A future standard will need to keep each domain and its timing explicit instead of collapsing safety into one surrogate.

Not all studies answer the same clinical question. Prospective human cohorts describe clinically apparent events and their short-term course, whereas longitudinal-use reports describe usability and interruption (Rubin et al., 2023; Mitchell et al., 2023; Davis et al., 2025; Greenspon et al., 2026). Retrieval, post-mortem and animal studies identify biological or material mechanisms that are usually unavailable during life (Bjånes et al., 2025; Woeppel et al., 2021; Szymanski et al., 2021). Standards and adjacent clinical literature provide reporting or procedural structure rather than BCI event rates (Eiber et al., 2021; International Organization for Standardization, 2026). The available evidence is enough to define what should be observed, but not to set universal treatment or explantation thresholds (Patrick-Krueger et al., 2025; Lim et al., 2025; Greenspon et al., 2026; International Organization for Standardization, 2026). Table 2 links each observation to the decision it can support, the remaining uncertainty and the element required in a future standard.

**Table 2:** Evidence-to-decision map for implanted-BCI safety assessment.

Safety decision	Current evidence	What it can support	What remains unresolved	Element needed in a standard
Clinical and procedural harm	BrainGate: *n* = 14, 12,203 participant-days; SWITCH: *n* = 4, 12 months (Rubin et al., 2023; Mitchell et al., 2023)	Event phenotype, timing, course and short-term outcome	Small platform-specific cohorts; non-equivalent denominators	Common event dictionary, time windows and participant-level recurrence
Chronic biological interface	980 electrodes from 10 explanted arrays in three people, plus one unimplanted control array; post-mortem findings (Bjånes et al., 2025; Woeppel et al., 2021; Szymanski et al., 2021)	Plausible tissue, material and vessel changes	Usually no contemporaneous human marker or rate	Architecture module and triggers for problem-directed investigation
Technical integrity and function	Five-year surface case and 7.5-year Utrecht use (Vansteensel et al., 2024; Davis et al., 2025); recording studies (Colachis 4th et al., 2021; Simeral et al., 2011)	Usability, drift and interruption over time	Performance change is not specific for injury	Versioned exposure, matched retest and participant consequence
Stimulation exposure	Five participants, 27 implant-years and >170 million ICMS pulses (Greenspon et al., 2026)	Tested parameters, cumulative exposure and observed effects	No universal safe dose or cross-target threshold	Delivered waveform, charge, duty cycle, contacts and cumulative exposure
Revision and end-of-use	Explant and loss-of-access analyses (Bublitz and Gilbert, 2023; Vooijs et al., 2025); post-trial work (Young et al., 2026; Higgins et al., 2026)	Removal, retention, support and access consequences	Sparse denominators and incomplete follow-up	Final-status documentation, fallback arrangements and responsible team

Evidence streams are complementary and do not provide directly comparable event rates.

## Why current evidence cannot yet be converted into a safety rule

### The number of independently implanted participants remains small

One participant or small programme cohort may generate several publications, including feasibility, algorithm development, home use and long-term follow-up. For example, the initial Utrecht report and its seven-year follow-up describe the same implanted participant (Vansteensel et al., 2024; Vansteensel et al., 2016). Counting each paper as a separate implanted sample therefore overstates independent human exposure. Reports should be linked at participant level before they are combined.

### Exposure denominators are inconsistent

Safety questions require different denominators. Participant count and participant-days describe people and observed time at risk; active-use hours describe actual use. BrainGate reports 68 device-related events in 14 participants over 12,203 participant-days, SWITCH reports eight effects in four implanted participants over 12 months, and the ICMS study adds pulse count and implant-years (Rubin et al., 2023; Mitchell et al., 2023; Greenspon et al., 2026). These quantities cannot be reduced to one incidence measure without participant-level recurrence and active-use exposure. Stimulation-hours and pulse count add electrical exposure, but neither replaces waveform and charge information (Greenspon et al., 2026; Eiber et al., 2021; Cogan et al., 2016). Interpretation is further affected by version changes, withdrawal and missed visits. Spontaneous-report databases such as MAUDE cannot supply incidence without reliable exposure denominators (U.S. Food and Drug Administration, 2025).

MAUDE can nevertheless identify safety signals during real-world use, including recurrent symptoms, device problems and delayed reporting. Awan et al. analysed 200 unique reports involving transcranial magnetic stimulation (TMS), describing symptom patterns, device issues and reporting delays (Awan et al., 2026). Although TMS is non-invasive and these findings do not establish implanted-BCI risk, the study illustrates the value of postmarketing surveillance in neurotechnology. Such signals can guide targeted investigation and prospective follow-up; report counts alone cannot establish causality, incidence or comparative device safety.

### Not observing a problem may reflect the limits of the method

A negative finding is meaningful only in relation to how it was obtained. Clinical examination detects manifested consequences; imaging is constrained by resolution, artefact and the permitted implant configuration (Boutet et al., 2020; Harwood et al., 2024; U.S. Food and Drug Administration, 2023). Device metrics are non-specific, and instability or recalibration can have several explanations (Colachis et al., 2021; Wilson et al., 2026; Pun et al., 2024; Swanson et al., 2026). Histology and material inspection usually become available only at retrieval or death and cannot reconstruct the entire time course (Bjånes et al., 2025; Woeppel et al., 2021; Szymanski et al., 2021). Absence of symptoms, normal imaging and signal stability do not resolve the same uncertainties.

### End-of-use, loss to follow-up and unsuccessful trajectories are under-described

The last successful demonstration says little about what followed once use stopped. Retrieval reports describe selected endpoints (Bjånes et al., 2025; Woeppel et al., 2021), while legal and ethical studies address removal and loss of access (Bublitz and Gilbert, 2023; Vooijs et al., 2025). Work on consent, post-trial support and abandonment identifies responsibilities that extend beyond active research (Young et al., 2026; Higgins et al., 2026; Okun et al., 2024). End-of-use status and the reason for missing follow-up would therefore be retained in the proposed safety record, even when no later performance data are available.

## From fragmented evidence to a BCI clinical safety assessment standard

Existing documents address separate parts of the problem. FDA guidance covers nonclinical testing and clinical-study considerations for implanted BCIs used to restore motor or sensory function (U.S. Food and Drug Administration, 2021). Neural-interface reporting recommendations and ISO 14155 address research transparency and good clinical practice (Eiber et al., 2021; International Organization for Standardization, 2026). Neurostimulator, active-implant and MR documents address particular device or use conditions (International Organization for Standardization, 2017; U.S. Food and Drug Administration, 2023; International Organization for Standardization, 2014). What remains absent is a prospectively validated BCI-specific pathway that links a longitudinal finding to verification, attribution, escalation, intervention and end-of-use across implant architectures.

The clinical findings and existing reporting requirements provide the basis for the framework below. Its combination of a common dataset, architecture-specific modules and an event-triggered pathway is proposed here for prospective evaluation. These components have not been validated as a BCI assessment standard and do not establish clinical action thresholds.

### A reporting standard, an assessment standard and a clinical guideline are different products

These three products should not be treated as interchangeable. Reporting standards define the information that must be retained. Assessment standards also specify when and how observations are made, how change is confirmed and who reviews it; clinical guidelines additionally link findings to recommended action after evidence grading and benefit–risk judgement. Current neural-interface and clinical-investigation standards support the first layer and parts of the second (Eiber et al., 2021; International Organization for Standardization, 2026; Sawyer et al., 2025). They do not yet justify universal BCI treatment, interruption or explantation rules. Keeping these products separate avoids presenting a complete-looking checklist as a validated clinical guideline.

On the evidence reviewed here, terminology, baseline state, actual configuration, versioned exposure, denominator choice, event course and final implant status can be harmonised now (Eiber et al., 2021; International Organization for Standardization, 2026). The same evidence does not support one visit schedule, imaging interval, stimulation-dose limit, interruption rule, or revision or explantation threshold across architectures (Patrick-Krueger et al., 2025; Lim et al., 2025; Rubin et al., 2023; Greenspon et al., 2026).

### Where a standard could improve implantation and postoperative safety

A future assessment standard would need a preimplant baseline that can distinguish disease progression from a new procedural or device-related change, together with a medication plan, imaging constraints and a tested fallback. The operative record would retain the exact hardware, target, access route, final position, fixation, verification imaging and any deviation from the planned procedure. Postoperative records would use defined windows for neurological, wound, infection, medication and device observations, while allowing immediate event-triggered assessment between scheduled visits (Rubin et al., 2023; Mitchell et al., 2023; U.S. Food and Drug Administration, 2021; Eiber et al., 2021). The record itself will not prevent a complication, but it can reduce avoidable variation in what clinicians check, document and escalate.

### A candidate common core organised around decisions

Table 3 converts those decisions into a candidate minimum dataset. The proposed core aims to limit reporting burden, although its feasibility across centres remains to be established. Architecture-specific modules would capture risks associated with the actual interface. Accordingly, the proposed module for percutaneous systems would include skin-route observations (Rubin et al., 2023). Fully implanted systems would include packaging, lead and power information (Bullard et al., 2020; Eiber et al., 2021; International Organization for Standardization, 2014). Surface systems would include extra-axial findings, fixation and removal considerations (Arya et al., 2013; Benabid et al., 2019). Venous systems would include patency and antithrombotic exposure (Mitchell et al., 2023; Oxley et al., 2021). Stimulation would add delivered settings and unintended effects (Greenspon et al., 2026; Flesher et al., 2016; Fernández et al., 2021; Cogan et al., 2016).

**Table 3:** Candidate minimum dataset organised by the safety decision supported.

Field	Minimum record	Decision supported	Architecture-specific addition
Participant and baseline	Disease course, neurological and functional state, clinical risks, medication, support and tested fallback	Attribute later change and plan perioperative risk	Relevant respiratory, skin, seizure and antithrombotic context
Procedure and configuration	Components and versions, target, access route, final position, fixation, verification and procedural deviation	Identify actual exposure and distinguish planned from unplanned change	Skin route, surface plane, venous segment, package and lead path
Time, use and stimulation exposure	Implant-days, observed follow-up, active-use hours, version intervals; delivered stimulation when applicable	Choose the correct denominator and relate change to exposure	Waveform, charge, contacts, duty cycle, pulse count and medication exposure
Repeated safety state	Neurology, wound, imaging, device, signal, telemetry, function, reliance and caregiver burden	Detect within-person trend and disagreement between domains	Interface-specific tissue, vessel, fixation, removal or power observations
Event pathway	Phenotype, severity, seriousness, relatedness, onset, duration, recurrence, event count, participants affected, detection, tests, treatment, responder and response time	Escalate, adjudicate and learn from the course	Event-specific clinical and technical investigations
Final status	Recovered use, reconfigured, revised, explanted, inactive retained, withdrawn, lost to follow-up or abandoned	Close the record and retain unresolved risk	Removal plane, retained components, fallback and post-trial support

Multicentre testing is required before adoption as an assessment standard.

FDA guidance, neural-interface reporting recommendations and ISO 14155 provide a basis for documenting baseline status, procedural details, device exposure, clinical events and device state (U.S. Food and Drug Administration, 2021; Eiber et al., 2021; International Organization for Standardization, 2026). Ethical and post-trial support literature informs the inclusion of participant interests, support arrangements and final implant status (van Stuijvenberg et al., 2026; Young et al., 2026; Higgins et al., 2026; Okun et al., 2024). The specific fields, including functional reliance and caregiver burden, and their assembly into this decision-oriented dataset are our proposed approach to the reporting gaps described above. Neither the exact field set nor its designation as a minimum has been established by formal consensus or prospective validation.

A hardware exchange, firmware update or decoder revision changes the configuration to which a participant is exposed. Start and stop dates and accumulated exposure are therefore included in the proposed record for each version rather than attaching one version label to the whole implant period (Bullard et al., 2020; Eiber et al., 2021; International Organization for Standardization, 2014). If exposure is not versioned, an event after an update may be assigned to the wrong configuration, and apparently stable long-term data may combine unlike systems.

### Combining scheduled surveillance with event-triggered assessment

Scheduled visits can reveal gradual trends, but clinically important changes may arise between them; event-only follow-up has the opposite weakness and misses silent drift. A candidate assessment standard would therefore define a minimum phase-specific dataset and categories of triggers rather than impose one visit frequency on every system (U.S. Food and Drug Administration, 2021; Eiber et al., 2021; International Organization for Standardization, 2026). New neurological, wound or systemic findings require timely clinical assessment, with urgency determined by the presentation (U.S. Food and Drug Administration, 2021; Pritzlaff et al., 2025). Within the proposed pathway, an isolated technical change would first be confirmed under matched conditions and would trigger clinical review if it persisted or remained unexplained (Eiber et al., 2021; Wilson et al., 2026; Pun et al., 2024; Swanson et al., 2026). This gives centres a common response structure without fixing treatment thresholds before they have been validated.

### Recording the course of an event

The label ‘infection’ or ‘seizure’ says little about an event’s course. Severity describes intensity; seriousness concerns outcomes such as hospitalisation, disability or death. The proposed record would include both, alongside relatedness, onset, duration, recurrence, detection method and device or exposure context (Rubin et al., 2023; Mitchell et al., 2023; Greenspon et al., 2026; Eiber et al., 2021). Tests, treatment, interruption of use, response time and outcome show how the episode was managed (Pritzlaff et al., 2025; International Organization for Standardization, 2026). For recurrence, the proposed record would include both event counts and the individual participant’s course.

### Evaluating whether the proposed standard improves care

A completed form does not show that the standard works. Relevant outcomes include missing data, agreement between assessors, time from trigger to clinical review, false alarms, missed clinically important events, changes in management, participant burden and consistency across centres. ISO 14155 provides a basis for independent event adjudication and monitoring (International Organization for Standardization, 2026), while BCI outcome workshops emphasise clinically meaningful consequences and trial design (Sawyer et al., 2026b; Sawyer et al., 2026a; Sawyer et al., 2025). Architecture-specific analyses should remain separate until pooled safety rates are justified.

Participants and caregivers could help determine which functional interruptions, support failures and follow-up burdens are clinically meaningful (van Stuijvenberg et al., 2026; U.S. Food and Drug Administration, 2022). The proposed dataset would include participant-level linkage, device and software version control, and a final status for every implant. Without these elements, recurrent events, overlapping publications and silent loss to follow-up will continue to distort the safety record.

### From a candidate standard to living clinical guidance

Table 4 separates the stages. Consensus can identify important fields when evidence is sparse, but an action threshold cannot be validated by consensus alone. A multidisciplinary process that includes lived experience can establish content validity (Eiber et al., 2021; van Stuijvenberg et al., 2026; U.S. Food and Drug Administration, 2022; Sawyer et al., 2025). Prospective multicentre data are then needed to test reliability, detection, burden and clinical consequences (International Organization for Standardization, 2026). Only after that step should graded recommendations for interruption, revision, explantation or return to use be issued. A predefined update process is essential because electrode design, software and patterns of home use will continue to change.

**Table 4:** Staged development from harmonised reporting to living implanted-BCI clinical safety guidance.

Stage	Product	Evidence to generate	Gate before progression
1	Harmonised reporting	Definitions, core fields, denominators, participant linkage and versioned exposure	Content validity and feasibility without untested action thresholds
2	Prospective assessment standard	Scheduled and event-triggered collection, architecture modules, agreement, adjudication, timeliness and burden	Reliable implementation and evidence that important change is identified consistently
3	Evidence-graded action guidance	Consequences of triggers, management changes, missed events, benefit–risk and participant priorities	Reproducible evidence supports interruption, restart, revision or explantation recommendations
4	Implementation and registry	Coverage, audit, interoperability, end-of-use capture and between-centre performance	Improved care without disproportionate participant or service burden
5	Living update	Registry outcomes, new architectures, hardware/software revisions and emerging surveillance tools	Predefined review cycle and version-controlled change

## Research outlook: detecting biological change below current clinical resolution

Clinical tools are relatively insensitive to early microscopic change. Blood biomarkers and multi-omic methods are therefore research targets outside the clinical core. Glial fibrillary acidic protein (GFAP) and ubiquitin C-terminal hydrolase L1 (UCH-L1) are supported by substantial literature in traumatic brain injury, but neither has a validated threshold for chronic BCI use (Korley et al., 2022; Hossain et al., 2024; Bazarian et al., 2025). Cell-free DNA methylation can provide tissue-of-origin information, including brain-derived DNA research (Moss et al., 2018; Loyfer et al., 2023; Gaitsch et al., 2023; Pellegrini et al., 2024). Tissue-injury and cell-free RNA studies show methodological feasibility elsewhere, not validation in BCI recipients (Zhang and Li, 2023; McNamara et al., 2025; Vorperian et al., 2022; Tu et al., 2026). Studies in BCI recipients would need repeated within-participant sampling linked to surgery, imaging, device state and clinical events; seizure, infection, systemic illness and disease progression would need to be treated as competing explanations. These markers should remain outside the clinical core until prospective human data establish their specificity, timing and clinical utility.

## Limitations

The available evidence does not support a safety ranking of implant architectures. Most human data still come from early feasibility studies or small programme cohorts, and the same participant may appear in several publications on function, home use or later follow-up (Patrick-Krueger et al., 2025; Lim et al., 2025; Rubin et al., 2023; Wagner et al., 2026). Event definitions, follow-up duration and ascertainment also vary across protocols. Pooling these reports would create a more precise-looking rate without a comparable denominator.

Small and partly overlapping cohorts also limit applicability beyond the studied devices, implantation routes and participant populations. Findings from one architecture cannot establish event rates, surveillance schedules or action thresholds for another without architecture-specific evidence.

Much of the biological interface is also beyond direct observation during life. Material and local tissue findings may become available only after retrieval or death, so explant and post-mortem series describe selected endpoints rather than a continuous biological history (Bjånes et al., 2025; Woeppel et al., 2021; Szymanski et al., 2021). Animal studies can identify mechanisms and possible blind spots, but not a human incidence estimate or treatment threshold. Similarly, the absence of a reported event in a small cohort means only that the protocol did not detect one; an uncommon or clinically silent process remains possible.

The proposed core dataset, escalation pathway and staged route remain testable proposals, not a completed standard. Formal consensus would first need to establish whether the fields are understandable and feasible across centres. Prospective studies would then have to measure missing data, agreement between assessors, false and missed triggers, time to clinical review, participant burden and effects on management or patient-relevant outcomes (Eiber et al., 2021; International Organization for Standardization, 2026). Hardware, software and patterns of home use will also change during that process. Until these questions are answered, the framework is better used to organise data collection and comparison than to define universal interruption, revision or explantation thresholds.

## Conclusion

The field does not lack lists of possible hazards. The harder problem is that studies use different definitions, observation windows and exposure denominators, so clinical, biological, technical and functional findings cannot readily be aligned over time. On that evidence, a universal event rate or treatment threshold would be premature. The same evidence does support a practical starting point: shared definitions, a longitudinal core record, architecture-specific additions, event-triggered review, independent adjudication and a documented end-of-use status. Applied from preimplant planning through surgery and long-term follow-up, this framework could reveal avoidable variation in practice and bring emerging problems to clinical attention earlier. It should become formal guidance only if prospective studies show that it improves care; expert agreement alone is insufficient.
